# Symbiosis Between *Epichloë* Fungi and *Bromus* Grasses: A Review of Current Knowledge and Future Directions

**DOI:** 10.3390/jof11110807

**Published:** 2025-11-13

**Authors:** Jorge A. Luna-Fontalvo, Oscar Balocchi, Oscar Martínez, Máximo Alonso, Enrique Ferrada

**Affiliations:** 1 Faculty of Agricultural and Food Sciences, Graduate School, Universidad Austral de Chile, Valdivia 5090000, Chile; jorge.luna@alumnos.uach.cl; 2 Faculty of Basic Sciences, Universidad del Magdalena, Santa Marta 470004, Colombia; 3 Faculty of Agriculture and Food Sciences, Animal Production Institute, Universidad Austral de Chile, Valdivia 5090000, Chile; maximo.alonso@uach.cl; 4 Faculty of Sciences, Microbiology and Biochemistry Institute, Universidad Austral de Chile, Valdivia 5090000, Chile; oscar.martinez@uach.cl; 5 Faculty of Agriculture and Food Sciences, Plant Production and Health Institute, Universidad Austral de Chile, Valdivia 5090000, Chile; enrique.ferrada@uach.cl

**Keywords:** endophytic fungi, *Epichloë*, *Bromus* spp., grass-fungus symbiosis, south american grasslands

## Abstract

*Epichloë* is a genus of endophytic fungi that forms systemic, vertically transmitted, and asymptomatic mutualistic associations with grasses in the subfamily Pooideae. These symbioses are non-pathogenic and are of considerable importance in agronomic and livestock systems due to their roles in enhancing host fitness under biotic and abiotic stress. Several studies have reported associations between *Epichloë* endophytes and species of the genus *Bromus*, a taxonomically complex group characterized by varying ploidy levels and frequent hybridization. Among its sections, *Bromopsis* includes the highest number of species naturally colonized by *Epichloë* fungi, while sections *Bromus* and *Ceratochloa* show lower infection rates. In South America, endophytes such as *E. pampeana*, *E. tembladerae*, *E. typhina*, and morphotypes of *Neotyphodium* spp. have been documented in species including *B. auleticus*, *B. brachyanthera*, and *B. setifolius*, where they appear to contribute to stress resilience. Although most findings originate from Argentina, significant knowledge gaps remain regarding the diversity and distribution of these endophytes in native *Bromus* species across the continent. This review synthesizes the current understanding of *Epichloë*–*Bromus* interactions, emphasizing their ecological and agronomic relevance, particularly in South America. Key factors influencing the establishment of these symbioses are examined, and future research directions are proposed to advance the study of these associations.

## 1. Introduction

Certain cool-season grasses are commonly colonized by endophytic fungi of the genus *Bromus* (Fr.) Tul. & C. Tul., formerly known in their asexual state as *Neotyphodium* [1,2]. These endophytes systemically infect host plants without causing visible damage or disease symptoms and are primarily transmitted vertically through seeds, ensuring persistence across plant generations [3]. However, *Epichloë*-grass interactions are diverse, encompassing mutualistic, parasitic, or antagonistic relationships depending on the host and environmental context [4]. The benefits conferred by endophyte colonization are often contingent upon host genotype and surrounding environmental conditions, as suggested by the host–endophyte–environment interaction model [5,6].

Historically, these endophytic fungi have garnered considerable interest due to their role in synthesizing bioactive alkaloids, which contribute to plant defense through a mechanism described as defensive mutualism [7]. Compounds such as peramines and lolines exhibit insecticidal or deterrent effects, notably protecting grasses against herbivorous insects like the Argentine stem weevil in *Lolium perenne* [8,9,10]. Conversely, some *Neotyphodium* species, such as *N. lolii*, produce tremorgenic alkaloids like lolitrem B, which adversely affect grazing animals by inducing neuromuscular disorders, including ryegrass staggers and ergot-like poisoning [11,12,13]. These dual roles underscore the agricultural significance of *Epichloë* endophytes, particularly in forage systems, where they mediate both plant resilience and potential toxicity risks [14,15].

Beyond alkaloid production, *Epichloë* endophytes enhance plant adaptation to environmental stresses through morphological, physiological, and biochemical modifications. These include increased root biomass, improved water-use efficiency via stomatal regulation, and the accumulation of protective metabolites such as soluble sugars, amino acids (e.g., proline), antioxidant enzymes, and secondary metabolites [16,17,18]. Such traits confer tolerance to drought, flooding, salinity, extreme temperatures, nutrient imbalances, heavy metal toxicity, and oxidative stress [19,20].

Advances in biotechnology have enabled the artificial inoculation of grasses with selected *Epichloë* strains, particularly in *Lolium perenne* and *Festuca arundinacea* cultivars. The method developed by Latch and Christensen (1985) [21] for endophyte inoculation has laid the foundation for the commercialization of enhanced cultivars in the United States, Australia, and New Zealand [3,22]. For instance, the commercial strain *E. coenophiala* (MaxQ) is widely used in Jesup tall fescue for livestock grazing in the U.S., while endophyte-enhanced ryegrass cultivars such as AR1, AR5, AR37, and NEA2 are commercially deployed in New Zealand and other regions for their efficacy against insect pests.

Given this success, it is of considerable interest to explore the potential of artificially inoculating native grasses of agronomic value, especially species undergoing domestication. Temperate regions host a rich diversity of Poaceae, including both native and naturalized species with high forage potential. Within this context, the genus *Bromus* (subfamily Pooideae), comprising between 100 and 400 species across seven taxonomic sections, plays a prominent role in temperate and cold environments worldwide [23,24,25]. This genus includes annual, biennial, and perennial species, many of which are taxonomically complex due to frequent hybridization [26]. From an agronomic standpoint, species from the sections *Bromopsis* (syn. *Pnigma*) and *Ceratochloa* are valued for their biomass production and nutritional quality [27,28,29,30].

Although *Epichloë*/*Neotyphodium* associations have been predominantly reported in the *Bromopsis* section, limited data are available on their presence in *Ceratochloa* and other sections of *Bromus*, suggesting a need for further investigation [24]. Comparative studies between endophyte-infected (E+) and uninfected (E−) *Bromus* species have revealed promising ecological and agronomic advantages, particularly in relation to stress tolerance [31,32,33,34,35]. However, the precise mechanisms through which these fungi mediate host responses and influence soil microbial communities remain insufficiently understood [36,37].

Considering the increasing environmental pressures imposed by climate change, *Epichloë* endophytes represent a valuable resource for improving grassland resilience and productivity. *Bromus* species could benefit from targeted bioprospecting efforts aimed at identifying native fungal symbionts with desirable agronomic traits. This review synthesizes the current state of knowledge on *Epichloë*–*Bromus* associations worldwide and highlights their ecological, physiological, and agricultural implications. It also outlines future directions for research, particularly in the context of sustainable grassland management and climate adaptation strategies.

## 2. *Epichloë* Endophytic Fungi

The genus *Epichloë* (Fr.) Tul. & C. Tul., along with its formerly recognized anamorph *Neotyphodium* Glen, C.W. Bacon & Hanlin, belongs to the phylum Ascomycota, family Clavicipitaceae. These fungi were previously classified under the genus *Acremonium*, section *Albolanosa* Morgan-Jones & Gams [38]. To resolve taxonomic ambiguities, Leuchtmann et al. (2014) [39] conducted a comprehensive realignment of the genus *Epichloë*, consolidating anamorphic *Neotyphodium* species as synonyms. This revision recognized 10 species typified by teleomorphs, 24 by anamorphs, as well as three subspecies, six varieties, and 25 hybrid species.

Collectively referred to as “epichloid” endophytes, these fungi are readily isolated on various culture media, including potato dextrose agar, corn meal malt extract agar, milk starch agar, sucrose malt yeast extract agar, and glucose yeast extract agar [40,41]. In culture, they typically form colonies with cottony, woolly, velvety, or radial morphologies, characterized by abundant aerial mycelium and coloration ranging from white and pale cream to tan [42,43].

Epichloid isolates exhibit variable growth rates, which can be used to differentiate morphotypes. Slow-growing strains require four to five weeks for full colony development at 23 ± 2 °C in darkness, whereas moderately slow-growing types develop within two to three weeks. Fast-growing epichloids can form mature colonies in as little as five to seven days [34,44].

**Figure 1:**
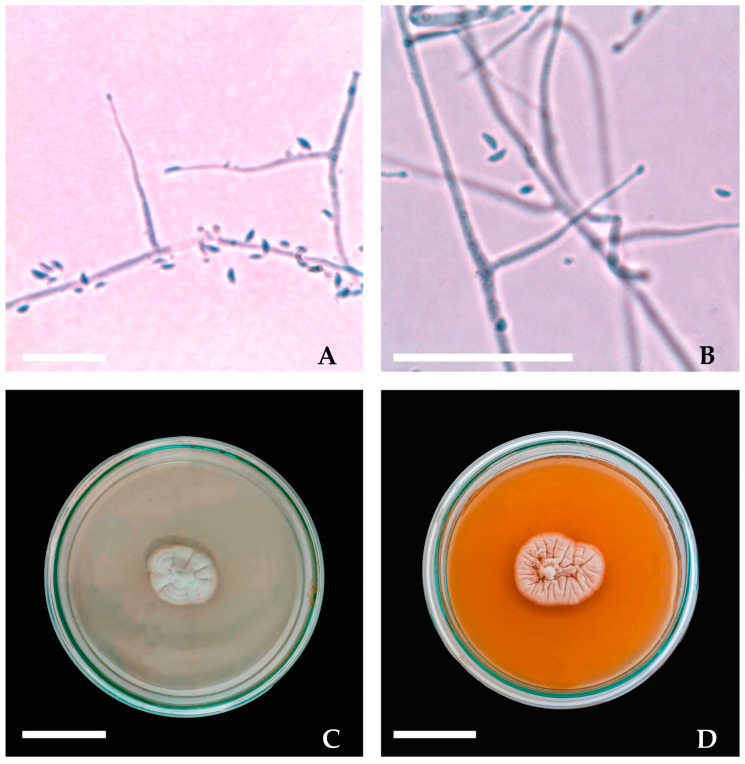
Photographs of *Epichloë uncinata* showing microscopic morphology and colony growth on culture media. Phialide and conidia were observed with aniline blue at 100× and 400× magnification (**A**,**B**). Colonies grown on corn meal agar and potato dextrose are 5 weeks old (**C**,**D**). Scale bars: 10 µm for phialide and conidia images, 50 mm for colony images.

Microscopically, *Epichloë* species exhibit enteroblastic conidiogenesis arising from hyaline phialides, which may occur solitarily, sympodially, or in branched forms. The resulting conidia show considerable morphological diversity, including allantoid, sigmoid, lunate, and limoniform shapes. Phialide and conidial dimensions are also variable. Some strains produce small conidia (4.5–7 µm) and phialides (12–18 µm), whereas others generate large conidia (8.5–13 µm) and exceptionally long phialides (60–90 µm). These morphological features are often associated with the specific host plant and the fungal growth rate [34,45]. As an example, morphological descriptions of *Epichloë uncinata* isolated from *Lolium perenne* are presented, including its in vitro growth on culture medium and its asexual microscopic structures (Figure 1).

In addition to their asexual characteristics, certain *Epichloë* species form sexual reproductive structures called stromata. These structures contain perithecia and colonize host inflorescences, often leading to spikelet abortion and partial or complete host sterility—a phenomenon known as “choke disease” [46]. Notably, stroma formation has been observed primarily in grasses native to the Northern Hemisphere [47].

The phylogenetic diversity of *Epichloë* species has been elucidated through analyses of molecular markers, including introns of the β-tubulin gene (tub2), translation elongation factor 1-α (tef1), actin (act1), and the internal transcribed spacer (ITS) region of rDNA [48,49,50]. These phylogenetic studies have revealed the evolutionary origins of *Epichloë* endophytes, highlighting both interspecific hybridization events and the presence of distinct, non-hybrid lineages. These lineages are closely linked to host specificity, morphological traits, and evolutionary history [42,51].

## 3. The Genus *Bromus*

The genus *Bromus* L., a member of the family Poaceae (subfamily Pooideae, tribe Bromaceae), comprises approximately 100 to 400 species [52,53]. These grasses are distributed widely across temperate and cold regions of both hemispheres, as well as in tropical montane zones [25]. The genus includes a diverse array of life forms—annual, biennial, seasonal, and perennial species—some of which exhibit winter dormancy [23]. Polyploidy and interspecific hybridization are major evolutionary forces shaping the genus *Bromus*, resulting in species with considerable variation in chromosome numbers and genome sizes [53]. Chromosome counts range from diploid (2n = 2x = 14) to highly polyploid forms (2n = 12x = 84), with a basic chromosome number of x = 7 [24]. Due to its high morphological plasticity, frequent hybridization, and polyploid complexity, the taxonomy of *Bromus* is notoriously challenging.

Flow cytometry analyses have made it possible to determine that nuclear DNA content correlates with the number of chromosomes in *Bromus* species. Thus, it has been determined that there are at least three categories within the genus based on DNA content —lowest group (11.43 to 12.65 pg/2C), middle group (21.45 and 22.77 pg/2C) and highest group (25.48 to 26.62 pg/2C) [54]. Likewise, Nizam et al. (2020) [54] indicated that cytogenetic analyses performed on *Bromus inermis* accessions showed that most were tetraploid with 2n = 4x = 28 chromosomes, while octaploid (2n = 8x = 56) and decaploid (2n = 10x = 70) accessions numbered 10 and 5, respectively.

Species classification is often hindered by overlapping characteristics. Traditionally, the genus has been divided into seven taxonomic sections or subgenera (Table 1), based on a combination of morphological, cytogenetic, and molecular traits. Key distinguishing features among sections include the number of veins on the glumes, spikelet morphology, shape of the lemma and awn (arista), karyotype composition, ploidy levels, genomic compatibility (mating systems), and serological markers [24,26]. In South America, 38 *Bromus* species have been recorded, distributed among five taxonomic sections. These include: *Bromopsis*, comprising 14 perennial species; *Bromus*, with nine introduced species; *Ceratochloa*, which includes eight species with both annual and perennial growth habits; *Genea*, represented by five introduced species; and *Neobromus*, which contains two native species [55]. This broad taxonomic diversity, coupled with the genus’s ecological adaptability, highlights *Bromus* as a key group for studies in plant evolution, forage development, and symbiotic associations such as those with *Epichloë* endophytes.

## 4. *Epichloë* Endophyte–Grass Interaction

A defining feature of epichloid endophytic fungi is their ability to establish symbiotic relationships with several cool-season grasses in the Poaceae family, notably *Lolium perenne* and *Festuca arundinacea* [2]. These associations have also been documented across other grass lineages, including members of the tribes Brachypodieae, Brachyelytreae, Bromeae, Meliceae, Poeae, Stipeae, and Triticeae [1].

**Figure 2:**
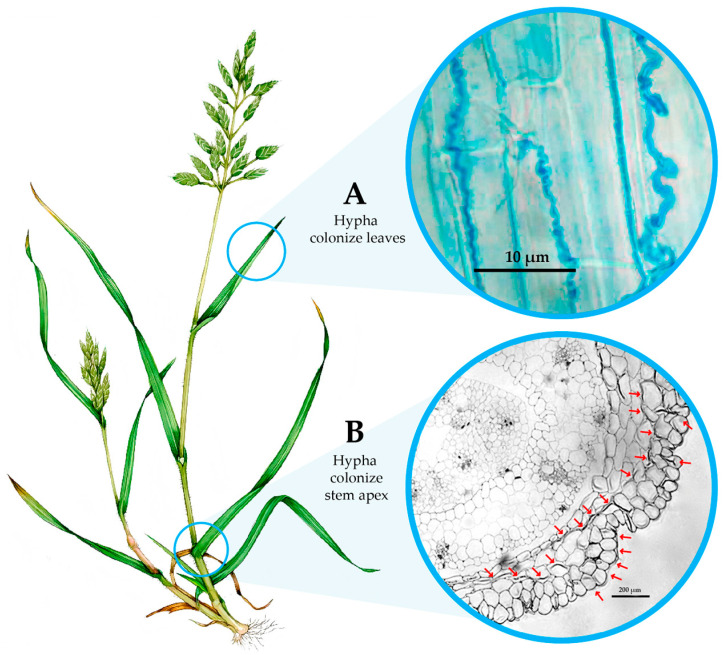
Schematic representation of colonization *Epichloë* in *Bromus* sp. (**A**) Microscope image of hyphae in leaf, stained with aniline blue. (**B**) Microscope image of stem apex, arrows indicate the intercellular position of hyphae Scale bars: 200 µm.

From an ecological perspective, the interaction between *Epichloë* spp. and their host grasses is typically non-pathogenic. The fungi systemically colonize foliar and stem tissues (Figure 2), particularly in axillary buds, without inducing visible symptoms. As the fungal hyphae progress through aerial parts—including leaves and reproductive structures—they ultimately reach and colonize the developing seeds. This enables vertical transmission from one generation to the next via hyphal penetration of the ovule and seed tissues [3].

Endophytic fungi are commonly classified based on their phylogeny and transmission strategy [56]. White (1988) [57] proposed a system that categorizes them into clavicipitaceous (Class 1) and non-clavicipitaceous (Classes 2, 3 and 4) groups. Class 1 fungi—such as epichloid endophytes—exclusively colonize grasses and are phylogenetically related. In contrast, non-clavicipitaceous endophytes colonize a broader range of hosts, including non-vascular plants, gymnosperms, and various angiosperms [38,58]. Class 1 *Epichloë*–grass associations have been further subdivided into three functional types based on reproductive and physiological traits [38,57]: Type 1 associations involve endophytes that regularly form stromata on host inflorescences, thereby suppressing sexual reproduction. These interactions are common in Poaceae and Juncaceae and often lead to “choke disease.”

Type 2 associations occur in endophytes that occasionally form stromata (1–10% incidence), primarily in species of the subfamily Festucoideae. These cases may lead to partial or complete sterility of the host plant, depending on whether inflorescence development is disrupted. Type 3 associations describe endophytes that do not form stromata but achieve high systemic colonization (>90%) without compromising host reproduction. Such associations have been documented in *F. arundinacea*, *F. versuta*, *L. perenne*, *L. temulentum*, *L. multiflorum*, *Stipa eminens*, and *S. robusta*. In these cases, the endophyte remains asymptomatic, floral structures are preserved, and vertical transmission through the seed is maintained—reflecting a stable, mutualistic relationship.

**Figure 3:**
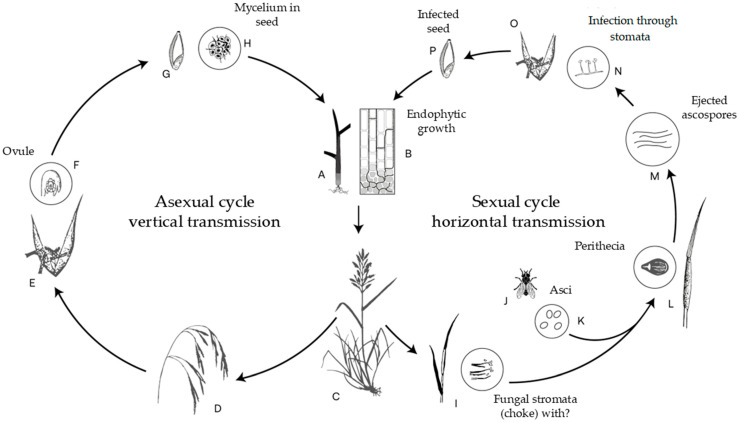
Reproductive cycle of the endophyte *Epichloë bromicola* on *Bromus benekenii*. In the asexual phase, the fungus grows and develops intercellularly (A), spreading through leaf sheaths (B), tillers meristems (C), continuing in inflorescences (D), infecting florets (E) and ovaries (F), until reaching developing seeds (G) and aleurone cells (H). In the sexual cycle, *E. bromicola* also occurs asymptomatically in leaf sheaths and meristems, then forms external stromata with conidia (I) inflorescences with strangling, the heterothallic phase is mediated by a fly (J) that transfers conidia of opposite mating (K), these give rise to the development of perithecia containing asci (L) and expelling filamentous ascospores (M), which germinate in repetitive cycles originating asexual spores (N) and mediate horizontal (contagious) transmission through infections of host flowers (O) and then on seeds (P). Adapted from Leuctmann and Schardl (1998) [59].

In *Bromus* species, *Epichloë* endophytes follow transmission dynamics like those described for *Festuca* and *Lolium*. For instance, *Epichloë bromicola* has been documented in *B. benekenii* and *B. ramosus* [59,60] (Figure 3). During the asexual phase, the fungus colonizes plant tissues intercellularly, beginning in the seed and extending into the leaf sheaths and eventually the inflorescences. The sexual phase initiates in the leaf sheaths without causing symptoms, but culminates in the formation of stromata on inflorescences, leading to their abortion—a process known as strangulation. Sexual reproduction in these heterothallic species is mediated by insect vectors, notably *Phorbia* spp., which transfer conidia between compatible mating types. Following fertilization, the fungus produces perithecia, asci, and ascospores within the stroma. These ascospores subsequently germinate and enter a cycle of asexual sporulation, facilitating horizontal transmission to new host individuals or populations.

### 4.1. Artificial Inoculation of Epichloë Endophytes in Forage Species

The toxicological issues arising from livestock consuming grasses infected with *Epichloë* endophytes have long been recognized, prompting advances in genetics and biotechnology aimed at manipulating and mutating *Epichloë* strains. These efforts focus particularly on detecting and modifying genes encoding alkaloid biosynthesis pathways [7,14,61].

Modern genetic and biotechnological improvement programs for forage grasses incorporate the artificial inoculation of endophytic fungi across populations. Due to their biotrophic nature, *Epichloë* endophytes can be isolated in pure culture and subsequently propagated to inoculate compatible host plants [62]. This approach has facilitated the successful transfer of endophytes between different genera and tribes of grasses, providing valuable insights into the symbiotic relationship and its agricultural potential [6,22].

A cornerstone technique for artificial inoculation is the cleavage inoculation method, originally described by Latch and Christensen (1985) [21]. This method involves germinating grass seeds on water agar under dark conditions to produce etiolated seedlings, into which *Epichloë* mycelium is introduced via a slit or wound at the mesocotyl–coleoptile junction or just below the first leaf on the coleoptile. This technique remains widely used both in technological applications and in comparative studies involving endophyte-inoculated plants [3,63].

Alternatively, Johnson-Cicalese et al. (2000) [64] developed a method involving mycelium insertion at the base of young or mature tillers, which has also yielded high infection rates. However, successful artificial inoculation depends heavily on genetic compatibility between the fungal endophyte and the host grass, as well as on the ability of the endophyte to establish vertical transmission to subsequent generations [7]. Infection levels in artificially inoculated plants can vary widely, ranging from high to poor infection rates, with occasional failure to establish infection altogether [21].

This technology has enabled the commercialization of forage seeds containing endophytes that produce alkaloids effective for pest control but non-toxic to livestock. Such advances have been implemented extensively in countries like New Zealand, Australia, and the United States [22]. Notable examples include the development of fescue and ryegrass cultivars harboring endophyte strains such as MaxQ (*Epichloë coenophiala*), AR1, AR5, AR37, and NEA2 (*Neotyphodium lolii*), which produce alkaloids like peramine, lolines, and epoxy-janthitrem [3,65]. AgResearch in New Zealand is recognized as a global leader in the formulation and commercialization of forage grass seeds inoculated with epichloid endophytes.

Recently, Zhang et al. (2025) [66] evaluated six methods for inoculating endophytes in *B. inermis*: longitudinal cutting of sterile seedlings, cutting of sterile seedlings, injection into sterile seedlings, seed soaking, seed perforation and soaking, and longitudinal cutting of seeds. Notably, the method of cutting sterile seedlings showed the highest infection rate (2.11%).

Overall, artificial inoculation methods represent a promising strategy to develop forage cultivars capable of meeting contemporary agrifood production challenges, including adaptation to biotic and abiotic stresses intensified by climate change [6]. Furthermore, these techniques hold significant potential for enhancing the agronomic performance of forage species such as those in the genus *Bromus*, where natural endophyte infection is limited to certain populations, thereby broadening the prospects for artificial inoculation to improve grassland productivity.

### 4.2. Response of Grasses Infected with Endophytic Epichloë Fungi to Biotic and Abiotic Stresses

*Clavicipitaceous* endophytic fungi, particularly those of the genus *Epichloë* and its anamorph *Neotyphodium*, have become increasingly important in agronomic research due to their mutualistic associations with cool-season forage grasses. These fungi systemically and asymptomatically colonize host tissues, conferring enhanced tolerance to both biotic and abiotic stressors. Notably, infected grasses exhibit increased resistance to insect herbivory, plant pathogens, drought, soil waterlogging, salinity, temperature extremes, ammonia toxicity, oxidative stress, and heavy metal contamination [2,3,15,67].

A key mechanism underlying herbivory resistance is the fungal production of bioactive alkaloids. *Festuca arundinacea* and *Lolium perenne* cultivars have been widely studied in association with *Epichloë*, revealing the synthesis of compounds such as peramines and lolines, which deter insect feeding. However, some endophyte-derived alkaloids—such as ergovaline and indole-diterpenes—are toxic to livestock, posing challenges for pasture-based animal production systems [7,14,68,69].

To address these concerns, biotechnological interventions have led to the development of forage cultivars inoculated with selected endophyte strains that produce non-toxic alkaloid profiles. For instance, *Neotyphodium lolii* strains AR1 and AR37—widely adopted in New Zealand, Australia, and the United States—synthesize peramine and janthitrems, respectively, instead of livestock-toxic compounds [3,6,65]. These alkaloids reduce insect oviposition, larval survival, and weight gain, thereby enhancing pasture resilience [14,70,71].

Recent studies have highlighted the diversity of secondary metabolites produced by *Epichloë* endophytes beyond traditional toxic alkaloids. These include compounds with antifungal, insecticidal, and phytotoxic properties, which could be harnessed for sustainable pest management. Consequently, future research should consider the full alkaloid spectrum, not just the well-characterized toxic compounds [13].

Alkaloid concentrations in endophyte-infected grasses are influenced by multiple factors, including the host genotype, plant developmental stage, endophyte strain, and specific tissue colonization patterns [71,72]. Seasonal fluctuations also occur, with higher alkaloid levels typically observed during spring and summer, and lower concentrations during autumn and winter [73,74]. Environmental variables, such as atmospheric CO_2_ concentration and soil nitrogen availability, can further modulate alkaloid synthesis [75,76].

Quantitative analyses have shown high loline concentrations in seeds, stems, and leaves—ranging from 50 to 2000 µg/g—while lower but still effective levels occur in roots [9]. Peramine concentrations as low as 0.1 µg/g have been shown to deter adult insects, with 10 µg/g effective against larvae [76]. Experimental comparisons between endophyte-infected and uninfected *Lolium* and *Festuca* plants have demonstrated both antibiosis (e.g., reduced larval growth due to N-formyl loline and N-acetyl loline) and antixenosis (e.g., feeding deterrence by peramine) [9,77,78]. In these studies, insect larvae showed reduced weight gain or failed to develop when feeding on infected grasses, particularly during fall and winter [9,79].

Given the ecological and agronomic benefits of endophyte-mediated resistance, ongoing research continues to explore the potential of native *Epichloë* strains for alkaloid production, pest suppression, and plant growth promotion in an environmentally sustainable manner [80,81,82,83].

One of the first laboratory trials evaluating pest deterrence in *Bromus* species was conducted by Siegel et al. (1990) [84], who investigated the content of peramine and ergovaline alkaloids in *Bromus anomalus* plants infected with *Acremonium starrii* and their effects on the survival of the grass aphid (*Schizaphis graminum*). Subsequently, Ball et al. (1997) [72] assessed the same host-endophyte association in relation to leaf consumption and oviposition by the Argentine stem weevil (*Listronotus bonariensis*). The presence of alkaloids in certain *Bromus* species has also been studied at the genetic level. For instance, genes associated with the biosynthesis of ergot alkaloids (EA), indole-diterpenes (IDT/LTM), lolines (LOL), and peramines (PER) have been identified in *B. laevipes* infected with *Epichloë typhina* and *E. cabralii* [85]. Guerre (2015) [12] noted that while *Epichloë* endophytes in *Bromus* may produce ergot alkaloids, limited information exists on their toxicity to livestock such as cattle and horses. This underscores the need for detailed analyses of alkaloid profiles and their toxicological effects following ingestion of endophyte-infected grasses.

Among the plant defense strategies conferred by endophyte infection, niche exclusion has been proposed for associations such as *Bromus setifolius*, *Festuca ovina*, and *Poa ampla*. In this mechanism, the endophyte forms a protective mycelial network on the surface of leaf blades [35,86], which may physically inhibit the entry of fungal pathogens [5]. Other mechanisms of disease reduction include direct effects such as competition and induced resistance to pathogens and insects, and indirect effects such as the emission of volatile organic compounds that deter pathogen vectors or stimulate antagonistic microbes [87].

Abiotic stress is a major limiting factor for plant productivity, inducing morphological, physiological, biochemical, and molecular alterations that reduce crop yields [15,17]. Water deficit, soil salinity, heat, and oxidative stress are particularly damaging, often acting synergistically to cause cellular injury and plant death [88,89,90].

Research on endophytic fungi in grasslands has highlighted their potential to enhance plant resilience under adverse environmental conditions [2,17]. However, most studies have concentrated on economically important species such as *Festuca arundinacea* and *Lolium perenne*. Tolerance to environmental stress in these species has been demonstrated through comparative assays using endophyte-infected (+) and endophyte-free (–) plants, considering both wild and cultivated genotypes and the effects of domestication [91]. These studies have provided insight into the agronomic relevance of endophytes, revealing improvements in yield-related traits and stress-responsive morpho-physiological characteristics. As a result, key mechanisms involved in stress evasion, tolerance, and recovery have been identified [19].

To better understand the role of endophytic fungi under drought conditions, research has focused on fundamental mechanisms such as drought evasion, tolerance, and recovery. In *F. arundinacea* and *L. perenne*, morphological and physiological adaptations that support efficient water use have been reported. These include extensive root systems that enhance water uptake from the soil, reduce transpiration losses, and promote water storage in plant tissues during dry periods [17,18,79,92].

Drought avoidance strategies in grasses often involve modifications to the root system. In ryegrass, endophyte colonization has been associated with increased length and thickness of root hairs, enhancing water absorption and dry matter accumulation. In contrast, *Festuca* species such as *F. arundinacea* and *F. pratensis* tend to develop thinner root hairs [93]. Another key strategy is stomatal closure, which reduces water loss via transpiration. Infected fescue plants exhibit altered stomatal behavior, although this response is less pronounced in ryegrass. This suggests a more rapid stomatal conductance in endophyte-infected fescue compared to uninfected plants [94,95,96,97].

These physiological responses are likely mediated by biochemical signals produced by endophytes, which may prime host tissues for early water-deficit responses [19]. Moreover, during dry periods, endophyte-infected grasses tend to retain higher water content in basal tiller tissues. This may be attributed to increased solute accumulation, reduced leaf conductance and transpiration, and the development of thicker cuticles [96,98].

Drought tolerance in grasses infected with epichloid endophytes involves a suite of physiological and biochemical adaptations that enable plant tissues to withstand water deficits. Most forage grasses employ multiple survival strategies under drought conditions. These include the accumulation and translocation of compatible solutes such as glucose, fructose, polyols, proline, mannitol, and amino acids, along with osmotic adjustment mechanisms that maintain cell turgor and wall elasticity [99,100,101,102,103]. Additionally, secondary metabolites, organic acids, sugars, plant hormones, antioxidant enzymes, and protective proteins such as dehydrins contribute significantly to drought stress mitigation [18].

A prominent example is the biosynthesis of epoxy-pyrrolizidine alkaloids, particularly lolines, which enhance osmotic potential and reduce the physiological impact of water stress. These responses are typically classified into strategies of avoidance, tolerance, or recovery under drought conditions [19,102,103,104]. For instance, *Lolium perenne* infected with *Neotyphodium lolii* exhibits elevated antioxidant production, which is considered a key mechanism for drought resistance by protecting meristematic regions and maintaining membrane integrity against reactive oxygen species (ROS) [105]. Similarly, comparative studies of endophyte-infected and uninfected *Festuca arundinacea* have shown that dehydrin accumulation in infected plants is associated with increased tiller survival and enhanced drought tolerance [106].

Grasses harboring *Epichloë* endophytes have also demonstrated tolerance to a range of abiotic stresses, including soil salinity and acidity [18,107,108,109], low temperatures [67,110], flooding [111,112], nutrient limitations, and heavy metal toxicity [20,113,114].

In the context of salt stress and acidic soils, endophyte-infected grasses have exhibited superior performance compared to uninfected counterparts. This improved tolerance is attributed to the endophyte’s regulation of ion homeostasis—particularly by limiting intracellular Na^+^ accumulation while promoting K^+^ uptake in shoot tissues, which helps maintain osmotic balance and cellular integrity. Additionally, enhanced uptake of essential nutrients such as phosphorus (P) and nitrogen (N) improves overall ionic regulation [18]. Endophyte presence is also associated with increased net photosynthetic rates and the accumulation of osmoprotectants like glycine betaine, which mitigate oxidative stress. At the anatomical level, modifications such as increased xylem and phloem area, enlarged vascular bundles, and thickened leaf veins, stem cortex, epidermis, and endodermis contribute to water conservation and sustained physiological function under saline conditions [115].

Contrasting tests with *Bromus inermis* plants inoculated with *Epichloë bromicola* (E+) and free of endophytes (E−) subjected to saline-alkaline stress have shown that metabolites positively regulated by endophyte inoculation are significantly enriched in the citrate cycle and ascorbate and aldarate metabolism, suggesting that symbiosis with endophytic fungi indirectly triggers the production of reactive oxygen species (ROS) through multiple metabolic pathways. Similarly, saline-alkaline stress tests showed that the host’s antioxidant system was activated after inoculation, and total antioxidant capacity increased significantly compared to non-symbiotic plants (E−) under mild stress, providing important clues to reveal the complex mechanism of plant-fungus symbiosis [66].

Cold tolerance has yielded mixed results. For example, in *L. perenne* infected (E+) or uninfected (E−) with *E. festucae* var. *lolii*, no significant differences in cold resistance were observed between isogenic lines subjected to freezing stress [116]. However, in non-isogenic plants or cold-tolerant hosts with high infection frequencies, the endophyte may contribute to increased resilience. Supporting this, *Achnatherum inebrians* seeds infected with *E. gansuensis* exhibited enhanced germination at 10 °C, associated with upregulation of genes involved in unsaturated fatty acid biosynthesis, alkaloid metabolism, and protein turnover [117].

The influence of *Epichloë* on waterlogging tolerance is also variable. In some *Poa* species, endophyte infection does not significantly affect biomass production under water stress [18]. Conversely, *Hordeum brevisubulatum* infected with endophytes showed improved flooding tolerance, likely mediated by increased proline accumulation, reduced electrolyte leakage, and decreased malondialdehyde levels—an indicator of lipid peroxidation [49]. Similarly, in *Festuca rubra*, the response to waterlogging appears to depend on the compatibility between host and endophyte [118]. Enhanced activity of catalase and peroxidase enzymes, as well as improved shoot and root development, contribute to increased photosynthetic capacity under such conditions [119].

Nitrogen (N) is a critical nutrient in agriculture, often limiting plant growth and productivity. The role of *Epichloë* endophytic fungi in enhancing grass resistance to nitrogen-deficient soils has been increasingly studied [18]. These endophytes modulate key metabolic pathways, including the regulation of glucose-6-phosphate dehydrogenase (G6PDH), an enzyme integral to the pentose phosphate pathway that supports abiotic stress tolerance. This regulation also influences antioxidant activity through glutathione, the NADPH/NADP^+^ ratio, and photosynthetic efficiency [120].

Recent investigations have highlighted improvements in nitrogen use efficiency (NUE) in plant-endophyte symbiosis. Infected plants exhibit elevated activities of nitrate reductase and nitrite reductase enzymes, coupled with increased levels of nitrate (NO_3_^−^) and ammonium (NH_4_^+^), critical intermediates for nitrogen assimilation. Moreover, glutamine synthesis is upregulated in *Epichloë*-infected (E+) plants, facilitating the conversion of inorganic nitrogen into amino acids [121]. Notably, G6PDH regulation appears essential for maintaining metabolic balance under low nitrogen conditions [120].

In a related study, *Epichloë gansuensis* infection of *Achnatherum inebrians* led to metabolic reprogramming, increasing the accumulation of organic acids, fatty acids, and amino acids within the host plant. The secretion of organic acids under nutrient stress enhances mineral solubilization and uptake from the soil, thereby supporting plant growth under nutrient-limited conditions [18].

Phosphorus (P) deficiency is another major constraint to plant productivity, often limiting the availability of other essential nutrients. Infection by *E. gansuensis* has been shown to improve tolerance to low phosphorus in *A. inebrians*, relative to uninfected plants. This tolerance is associated with endophyte-mediated regulation of specific amino acid and organic acid metabolisms, which in turn enhance nutrient metabolism and photosynthetic performance [122].

Heavy metal contamination in soils poses a significant threat to plant physiological and metabolic functions. Multiple studies have demonstrated that perennial ryegrass (*Lolium perenne*) infected with *Epichloë* exhibits increased tolerance to cadmium (Cd), copper (Cu), and zinc (Zn) stresses, unlike endophyte-free counterparts [123,124]. Under heavy metal stress, *Epichloë*-infected plants show enhanced biomass production, increased tiller density, greater plant height, and elevated chlorophyll a and b content. Additionally, these plants exhibit a superior capacity to bioaccumulate Cu and Cd in their leaves compared to uninfected plants [125].

In *F. arundinacea* infected with *Epichloë* present in cadmium-contaminated soils, the content of chlorophyll a, chlorophyll b and carotenoids shows significant variations in response to different CO_2_ concentrations, soil types and the presence or absence of cadmium. For example, in clay soils under high CO_2_ concentrations, chlorophyll a and b values of 0.45 mg g^−1^ and 0.37 mg g^−1^ were recorded, respectively. Meanwhile, carotenoid content reached values of 1.15 mg·g^−1^ in sandy loam soil [114].

Other studies, such as that by Shi et al. (2024) [126], demonstrate the capacity generated by *Epichloë bromicola* in *Elymus dahuricus* under conditions of stress due to Cd contamination in mining soils. It was observed that Cd treatment negatively influences plant growth; however, E+ plants grew as well as or better than E− plants. It was also determined that Cd increases the production of proline and antioxidant enzymes in E+ plants, and that *E. bromicola* has a beneficial effect by improving plant growth and antioxidant capacity.

Conversely, in the context of zinc stress, infected grasses display reduced Zn accumulation in leaves while maintaining higher tiller numbers and total biomass. This reduced leaf Zn concentration may mitigate toxic effects on photosynthesis, thereby contributing to improved growth performance [113].

A crucial physiological adaptation under heavy metal stress involves enhanced antioxidant enzyme activity. These antioxidants regulate osmotic balance, protect photosynthetic machinery, and alleviate oxidative damage caused by metal-induced reactive oxygen species [127].

## 5. *Bromus* Species Associated with Endophytic Fungi *Epichloë*

In recent years, associations between *Bromus* species and *Epichloë* (formerly *Neotyphodium*) endophytic fungi have attracted growing scientific interest. These symbioses appear most frequently in species belonging to the section *Bromopsis*, with fewer reports in the sections *Bromus* and *Ceratochloa* [24]. Host plant ploidy levels are closely linked to endophyte compatibility and infection rates: diploid *Bromus* species tend to be more susceptible to colonization by epichloid endophytes than polyploid or hybrid-derived plants. Moreover, genetic manipulation and seed improvement efforts may partly explain differences observed between natural and cultivated *Bromus* populations in establishing endophyte interactions [24,128].

Table 2 summarizes *Bromus* species known to form associations with endophytic *Epichloë* fungi, alongside their global geographic distribution. Numerous studies highlight the ecological and agronomic potential of these endophytes within natural grasslands containing *Bromus* species [28,129,130,131,132,133].

Worldwide, especially in South America, epichloid endophytes have been detected in several species of the *Bromopsis* section, including *B. auleticus*, *B. brachyanthera*, *B. setifolius*, *B. benekenii*, *B. erectus*, *B. ramosus*, *B. tomentellus*, and *B. anomalus* [129,130,134]. Iannone et al. (2011) [34] further support the hypothesis that the sexual stage of *Epichloë* fungi is absent in the Southern Hemisphere, which corresponds with a high diversity of asexual endophytes in South America.

Monitoring natural populations remains critical for understanding the prevalence and impact of these plant-fungus associations. Such studies inform breeding programs by highlighting the role of *Neotyphodium* endophytes in conferring resistance to biotic and abiotic stresses in grasses [133,135,136].

**Table 2:** Global review of associations between *Bromus* species and endophytic *Bromus* fungi.

Section	*Bromus* Species	Endophytic Fungus	Country	Source
*Bromopsis* (*Pnigma*)	*B. auleticus*	*Neotyphodium* sp.	Argentina, Brazil, Uruguay	[30,33,37,133,137,138,139,140,141]
		*Bromus pampeana* (*N. pampeanum*)		
		*E. tembladerae* (*N. tembladerae*)		
	*B. brachyanthera*	*Neotyphodium* sp.	Argentina, Brazil, Uruguay	[34,134]
	*B. setifolius*	*N. tembladerae*	Argentina, Brazil, Uruguay	[34,36,130,131,134]
		*Neotyphodium* sp.		
	*B. setifolius* var. *pictus*	*E. tembladerae*	Argentina—Chile	[134]
		*E. typhina* subsp. *poae*		
	*B. auleticus*, *B. brachyanthera*, *B. setifolius*	*N. tembladerae* (*E. typhina* × *E. festucae*)	Argentina	[2,139]
		*N. pampeanum* (*E. typhina* × *E. festucae*)		
		*Neotypodium* sp. (non-hybrid)		
	*B. benekenii*	*E. bromicola* *Acremonium* sp.	France, Switzerland, USA	[2,32,129,142,143,144,145,146]
	*B. erectus*			
	*B. ramosus*	*Acremoniun typhinum* var *typhinum*	England	[147]
	*B. laevipes*	*E. typhina* subsp. *poae*	USA	[85]
		*E. cabralii*		
		*Bromus* sp. BlaTG-3		
	*B. anomalus*	*N. starii*	USA	[40,148,149]
		*A. coenophialum*		
		*A. lolii*		
		*Acremoniun* spp.		
	*B. inermis*	HMA—DSE	Canada	[150]
*Bromus*	*B. tomentellus*	*Neotyphodium* sp.	Iran	[151]
	*B. japonicus*	*E. elymi*	China	[66]
*Ceratochloa*	*B. catharticus* (*B. wildenowii*)	*Neotyphodium* sp.	Argentina	[152]

Conversely, several *Bromus* species have been identified as endophyte-free, including *B. brevis* Nees and *B. catharticus* var. *rupestris* (section *Ceratochloa*), *B. japonicus* Thumb (section *Bromus*), *B. madritensis* L. (section *Genea*), and *B. pellitus* Hack. (section *Bromopsis*) [34,128]. Additionally, Leyronas and Raynal (2001) [143] reported endophyte-free seeds in multiple species of section *Bromus* (e.g., *B. arduennensis*, *B. arvensis*, *B. asper*, *B. commutatus*, *B. hordeaceus*, *B. madritensis*, *B. racemosus*, *B. secalinus*), some from sections *Genea* and *Ceratochloa*. However, Colabelli et al. (2007) [152] documented a low infection rate (~16%) of *Neotyphodium* sp. in *B. catharticus* germplasm conserved at INTA-Argentina.

South American grasslands are known for their remarkable diversity of grasses, including many native species of significant agronomic value widely used in livestock production across Argentina, Brazil, Chile, and Uruguay. The genus *Bromus* is particularly notable, including species such as *B. auleticus*, *B. burkartii*, *B. catharticus*, *B. setifolius*, *B. stamineus*, *B. valdivianus*, and *B. wildenowii* [133,153].

In southern Chile, *B. catharticus* Vahl, *B. stamineus* Desv., and *B. valdivianus* Phil. (all from section *Ceratochloa*) are frequently confused, leading some researchers to consider them synonyms [153,154]. Among these, *B. valdivianus* stands out as a native perennial species of livestock importance, valued for its nutritional quality and notable drought tolerance during summer [29,155]. Despite these attributes, knowledge gaps remain regarding its interactions with endophytic fungi. Furthermore, *B. stamineus* (considered synonymous with *B. valdivianus*) and *B. catharticus* have not been found to associate with *Epichloë* endophytes [37,156]. However, in Chile, endophytes such as *E. tembladerae* and *E. typhina* have been identified in association with *B. setifolius* var. *pictus* [134].

### Functionality of Epichloë Endophytes with Bromus Species

Numerous studies have emphasized the significant benefits conferred by *Epichloë* endophytic fungi to their host plants [3,157,158]. A remarkable diversity of epichloid endophytes associated with various *Bromus* species has been documented, exhibiting a range of functionalities including protection against pathogens, enhancement of plant growth, biological control, and increased tolerance to environmental stresses (Table 3). These beneficial effects are often mediated through strategies such as defensive mutualism and antagonism toward plant pathogens. Nonetheless, uncertainties remain regarding the precise mechanisms governing these interactions, especially under specific environmental variables such as rainfall patterns, low temperature regimes, and geographical influences. Such factors may affect the stability and persistence of the symbiosis, as well as influence endophyte-mediated modulation of the rhizosphere microbiome and its role in nutrient mobilization [37,141].

To evaluate the agronomic and ecological dynamics of *Epichloë*-*Bromus* associations, researchers have employed a variety of approaches including greenhouse experiments, field trials, and in vitro bioassays. This multifaceted methodology facilitates a comprehensive understanding of plant-endophyte interactions across diverse environmental and biological contexts.

Particularly notable are findings from South America, where *Epichloë* endophytes associated with native perennial forage grasses such as *B. auleticus* and *B. setifolius* have been extensively studied. These species, widely distributed across Argentina, Brazil, Chilean Patagonia, and Uruguay, serve as key models for exploring the ecological and agronomic potential of these symbioses [35,37,138,139,149]. Additional investigations have been reported from Switzerland [32] and the United States [160].

Despite these advances, further research is warranted to explore the potential benefits and functionalities of *Epichloë* endophytes in other *Bromus* species, particularly those currently undergoing domestication for agronomic purposes. Such efforts would broaden the understanding of endophyte-mediated improvements in grass productivity and resilience.

## 6. Future Perspectives

Research on epichloid endophytic fungi in cool-season herbaceous plants of the Pooideae family has been extensive, highlighting their significant benefits, including enhanced crop yields and increased resistance to biotic and abiotic stresses. Within the genus *Bromus*, studies have predominantly focused on species belonging to section *Bromopsis*, while knowledge remains limited for sections *Bromus* and *Ceratochloa* (Table 2). Given that many *Bromus* species possess considerable agronomic value and are widely distributed across grasslands in the Americas, Eurasia, Africa, and Oceania [24], it is essential to expand research efforts to other *Bromus* species and rigorously evaluate the ecological and agronomic potential of their endophytic *Epichloë* associations.

**Figure 4:**
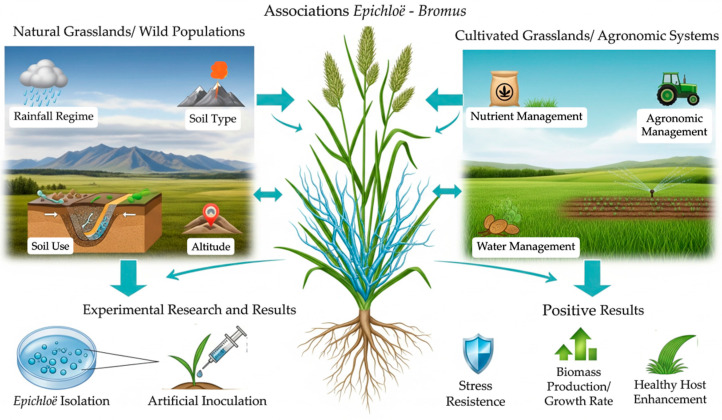
Schematic representation of factors influencing the study of endophytic *Epichloë* fungi in *Bromus* prairies.

Future research on *Epichloë* endophytes in *Bromus* species should carefully consider environmental variables such as grassland type, species origin (wild, introduced, or domesticated), geographic and climatic conditions, soil characteristics, and agronomic management practices (Figure 4). Incorporating these factors could deepen understanding of the mechanisms and strategies underlying plant-fungus interactions. This is particularly pertinent for polyploid hybrid *Bromus* species, whose complex genetics may influence their compatibility and symbiotic establishment with epichloid endophytes—a topic warranting further investigation [128].

Moreover, building on the hypothesis proposed by Iannone et al. (2011) [34], which suggests a high diversity of asexual endophytic fungi (*Neotyphodium*) in natural *Bromus* populations in South America, there is a clear need to intensify studies of endophytic fungi within this geographical region. Identifying and characterizing potential strains could provide valuable inoculum sources for agronomic applications. Such endeavors will substantially enhance our understanding of endophytic fungi from both ecological and agricultural perspectives, particularly regarding native, wild, and domesticated *Bromus* species.

## 7. Conclusions

A comprehensive analysis of the literature highlights the associations between endophytic fungi of the genus Epichloë and species of the genus Bromus, particularly in the section Bromopsis due to their genetic compatibility. The importance of this symbiosis for grasslands of agronomic interest is underscored, as the biochemical and physiological mechanisms involved optimize plant resources, thereby improving their adaptability to biotic and abiotic stress scenarios. Despite its relevance, research on this symbiosis in native South American species is limited. Therefore, future research should focus on identifying new symbiotic endophytes to develop more resistant forage cultivars, which will contribute to the sustainability of livestock systems in the face of climate change.

## Figures and Tables

**Table 1:** Sections of the genus *Bromus*, Adapted from Armstrong (1991) [23] and Williams et al. (2011) [24].

Tzvelev (1976) 7 Genera	Stebbins (1981) 7 Subgenera	Smith (1985) 7 Sections	Approximate Number of Species
*Anisantha*	*Stenobromus*	*Genea*	7
*Bromus*	*Bromus*	*Bromus*	30–40
*Bromopsis*	*Festucarja*	*Pnigma*	60
*Ceratochloa*	*Ceratochloa*	*Ceratochloa*	10–16
–	*Neobromus*	*Neobromus*	2
*Boissiera*	*Boissiera*	*Boissiera*	1
*Nevskiella*	*Nevskiella*	*Nevskiella*	1
*Littledalea*			

**Table 3:** Beneficial effects of endophytic *Epichloë* fungi on *Bromus* species.

Host Species	Endophyte	Bioactivity	Strategy or Mechanism of Action	Determination	Country	Source
*B. auleticus*	*Bromus* sp. *N. pampeanumtem* *N. bladerae*	Protection against the pathogen *Ustilago bullata* and promotion of plant growth.	Unknown	Bioassay—field	Argentina	[33,35,141]
	*E. pampeana*	Endophytes promote alterations in the host environment, regulate the diversity and abundance of rhizospheric fungi and arbuscular mycorrhizae, and contribute to the solubilization of phosphorus.	Unknown	Bioassay—greenhouse	Argentina	[36,37,140]
	*N. tembladerae*	Antiherbivory of *Spodoptera frugiperda*. Deterrent effect by the presence of ergovaline and peramine alkaloids.	Defensive mutualism	In vitro bioassay	Argentina	[149]
	*N. pampeanum* *N. tembladerae*	Biomass increase and loline production.	Mutualism	Bioassay—greenhouse—field	Argentina	[132]
*B. anomalus*	*N. starii*	Biological control by feeding and oviposition avoidance of *Listronotus bonariensis*.	Defensive mutualism	In vitro bioassay	Argentina	[159]
*B. setifolius*	*Neotyphodium* sp.	The presence of endophytes in the plant shows a positive correlation with various environmental factors, such as annual precipitation, cold temperatures, altitude, and geographical longitude. However, when environmental conditions are unfavorable for grass growth, this association is affected and there is a decrease in endophyte infection.	Unknown	Field	Argentina	[131,132]
*B. erectus*	*E. bromicola*	High concentrations of the endophyte improve the development and biomass of the plant during its vegetative phase. In addition, high levels of CO_2_ promote the antagonism of the endophyte towards the host.	Mutualism—antagonism	Bioassay—field	Suiza	[31]
*B. laevipes*	*Epicloë* sp./*Neotyphodium* sp.	The interaction between the endophyte and the grass improves tolerance to drought stress and broadens the geographical distribution range of the species. This interaction could respond to the demands imposed by climate change.	Mutualism	Field—greenhouse	USA	[160]
*B. benekenii*	*Neotyphodium* strain DEB 9701	Increase in host grass yield (expressed as aerial dry matter) under conditions of intraspecific competition.	Mutualism	Greenhouse	Switzerland	[32]
*B. inermis*	*E. bromicola*	Improves soil resistance to saline-alkaline stress by increasing the antioxidant capacity of plants	Mutualism	In vitro bioassay	China	[66]

## Data Availability

No new data were created or analyzed in this study. Data sharing is not applicable to this article.
